# Acute Uric Acid Nephropathy following Epileptic Seizures: Case Report and Review

**DOI:** 10.1155/2019/4890287

**Published:** 2019-02-04

**Authors:** Chinmay Patel, Caitlin P. Wilson, Naveed Ahmed, Yousef Hattab

**Affiliations:** ^1^University of Pikeville, Kentucky College of Osteopathic Medicine, Division of Nephrology, Pikeville Medical Center, USA; ^2^Family Practice Residency Program, Pikeville Medical Center, USA; ^3^Neurology Program, University of Pikeville, Kentucky College of Osteopathic Medicine, Pikeville Medical Center, USA; ^4^Division of Pulmonary and Critical Care Medicine, Pikeville Medical Center, USA

## Abstract

Acute hyperuricemia most commonly occurs in patients who experience tumor lysis syndrome. Hyperuricemia along with other electrolyte abnormalities like hyperkalemia, hypocalcemia, and hyperphosphatemia leads to acute kidney injury (AKI) due to acute uric acid nephropathy which is associated with significant morbidity. High risk patients are thus closely monitored for signs of these laboratory abnormalities. Extreme exercise, rhabdomyolysis, and seizures are rare causes of acute hyperuricemia. Serum uric acid level is not routinely monitored as a part of postictal labs. We report an unusual case of AKI in a young male with recurrent seizures and no associated rhabdomyolysis who was found to have acute uric acid nephropathy. Timely administration of Rasburicase prevented the need for dialysis in this patient and led to complete renal recovery. This case illustrates the importance of doing a urine microscopy and checking uric acid level in patients with recurrent seizures who develop unexplainable AKI, as timely management helps improve outcome. We also briefly review the pathophysiology of seizure related hyperuricemia and acute uric acid nephropathy.

## 1. Introduction

Uric acid is the final end product of purine metabolism. Acute hyperuricemia is caused by the increase of purine metabolism, which is the result of the increased cellular turnover or the aggressive cancer chemotherapy regimens which cause cell lysis and release of purine metabolites [1]. The deposition and accumulation of uric acid in renal tubules leads to acute uric acid nephropathy typically characterized by oliguric AKI, elevated serum uric acid concentration (usually more than 10-15 mg/dL), presence of uric acid crystals in the urinary sediment, and an elevated urine uric acid to creatinine ratio of greater than 1 [2]. Acute uric acid nephropathy is readily recognized when acute kidney injury (AKI) develops in cancer patients either due to spontaneous tumor lysis or following chemotherapy. Seizures seldom cause acute hyperuricemia and acute uric acid nephropathy and thus its diagnosis may be missed or delayed in this uncommon presentation. With timely recognition and management, the pathologic features of acute uric acid nephropathy are reversible. We report a patient who developed AKI due to acute uric acid nephropathy following recurrent seizures. Timely therapy with Rasburicase prevented the need for renal replacement therapy in our patient and resulted in complete renal recovery.

## 2. Case Presentation

**Figure 1:**
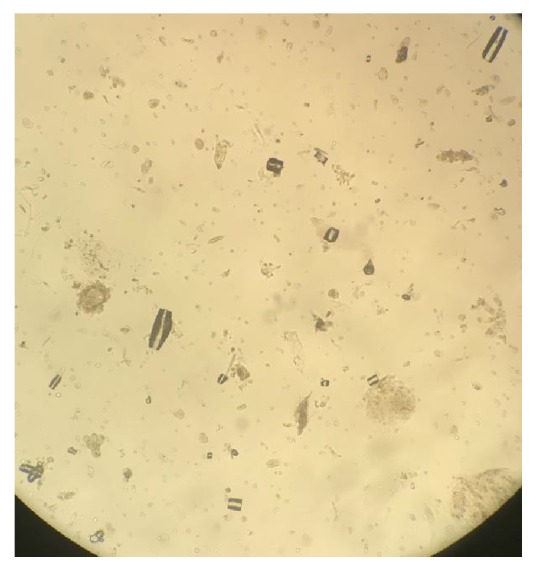
Urine microscopy under low power magnification showing uric acid crystals.

**Figure 2:**
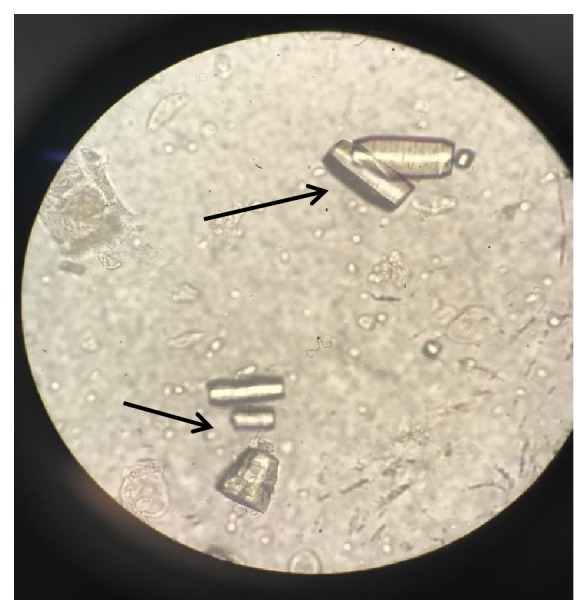
Barrel shaped uric acid crystals under high power magnification.

**Figure 3:**
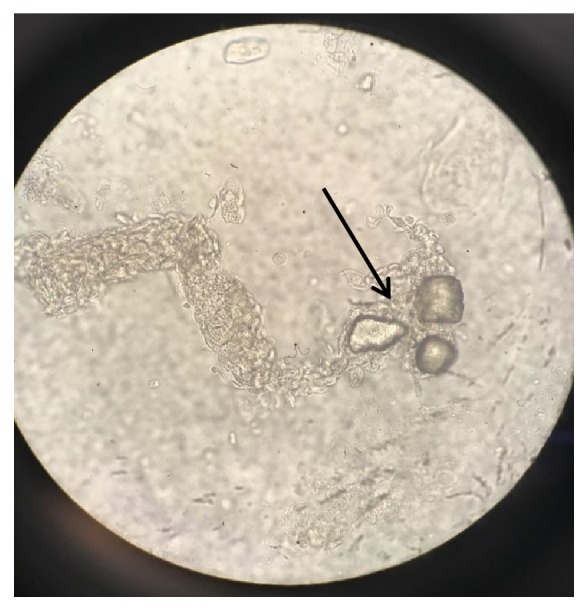
Diamond shaped uric acid crystals embedded in granular cast under high power magnification.

A 28-year-old white male with past medical history of epilepsy and asthma was referred to us from an outside hospital for recurrent seizures and higher level of care. EMS was called to his home after family reported him having two or three episodes of generalized tonic clonic (GTC) seizure like activity for an undocumented duration of time. EMS noted the patient was in a postictal phase and he was then taken to the nearest hospital where patient had another episode of GTC seizures lasting about 5-10 mins. Patient also became apneic and cyanotic and was subsequently sedated and intubated for airway protection. Levetiracetam loading dose was given. Brain CT done was unremarkable. Vitals signs showed elevated blood pressure of 170/90 and heart rate in 120s. Initial labs showed mild leukocytosis and a normal renal function with serum creatinine (scr) of 1.3 mg/dL. Urine drug screen was negative. He was then transferred to our hospital for neurology consultation and ICU admission. Laboratory test results are shown in Table 1. Repeat labs showed mild elevation in scr of 1.7mg/dL. Initial serum electrolytes and lactic acid were within normal limits. Serum creatine kinase (CK) level was mildly elevated at 297 U/L. Pt was started on iv hydration with ringers lactate (LR) at 100 ml/hr along with antiseizure medications, namely, iv Midazolam, Levetiracetam, and Lacosamide. He remained seizure free. Labs on day 2 showed worsening AKI with scr of 4.9 mg/dL and bicarbonate of 18 mEq/L with CK level of 663 U/L. Repeat scr was noted to be 5.2mg/dL with lactic acid of 3.6 mmol/L and phosphorus of 5.4 mg/dL. Patient had remained nonoliguric with urine output of about 40-60 ml/hr. Urgent urine microscopy was performed which showed numerous uric acid crystals and occasional granular casts [Figures 1, 2, and 3]. A stat serum uric acid level was noted to be significantly elevated at 15 mg/dL. Urinalysis showed clear urine with ph 5.5, specific gravity of 1.005, 0-5 RBCs, 0-5 WBCs, no proteins, and 1+ blood. A spot urine uric acid to creatinine ratio was found to be elevated at 1.2. Based on the above findings a diagnosis of acute uric acid nephropathy was made. IV fluids were changed from LR to iv sodium bicarbonate at 150ml/hr. Patient was given a dose of iv Rasburicase 0.2 mg/ kg. Repeat labs done about 4 hrs after Rasburicase administration showed that serum uric acid level had dropped to 4 mg/dL. Scr however continued to rise to 6.1mg/dL with CK level of 464 U/L. A significant increase in urine output of about 100-150 ml/hr was however noted soon after Rasburicase administration. Day 3 scr started trending down to 6 mg/dL and serum uric acid had further dropped to 0.8. There was continued renal recovery with scr of 5.5 and CK level of 792 U/L on day 4. Patient remained seizure free throughout his hospital stay and his renal function completely recovered to normal by day 7.

## 3. Discussion

Uric acid is the final oxidation product of dietary and endogenous purine metabolism. Uric acid must be continuously excreted to prevent its toxic accumulation in human tissues in which it is poorly soluble, especially in the acidic environment of the distal nephron. Approximately 75% of daily uric acid is excreted by the kidney [2]. Hyperuricemia is caused by states of enhanced purine catabolism which increases the urate load on the kidney leading to their intrarenal precipitation. Tumor lysis syndrome (TLS) is one such potentially life-threatening complication that occurs in high risk cancer patients with highly proliferative malignancies and large tumor burdens receiving cytotoxic chemotherapy. Lysed cancer cells release phosphorus, potassium, and nucleic acids which are metabolized to xanthine and then oxidized to uric acid. Patients at the highest risk of TLS are thus closely monitored for these laboratory abnormalities and started on appropriate preventive measures to reduce risk of acute uric acid nephropathy.

Acute hyperuricemia plays a major role in the pathogenesis of uric acid nephropathy. Supersaturation of urine and precipitation of uric acid crystals cause intraluminal obstruction of the distal nephron. This in turn leads to dilatation, inflammation, and obstruction of the proximal tubules [3]. Ejaz et al. have also described at least five crystal independent mechanisms by which uric acid contributes to AKI [4]. It induces renal vasoconstriction through direct inhibition of the endothelial nitric oxide synthase causing reduction in nitric oxide through stimulation of the renin-angiotensin system. Inflammatory pathways by which uric acid causes AKI include activation of proinflammatory mediators like monocyte chemoattractant protein-1, C reactive protein, and mitogen-activated protein kinase. In addition, uric acid also stimulates the production of oxidants via an increase in nicotinamide adenine dinucleotide phosphatase oxidase in both adipocytes and endothelial cells. Uric acid also has been found to have antiangiogenic properties by causing inhibition of endothelial migration and proliferation and inducing endothelial cell apoptosis. Finally, hyperuricemia leads to development of preglomerular arteriolar disease which impairs the renal autoregulatory response. Uric acid has also been reported to have antioxidant properties and it is possible that, in conditions of severe oxidative stress, a rise in uric acid might provide some antioxidant benefit [4, 5]. Ejaz el al. proposed that although the antioxidant properties of uric acid may be beneficial under certain conditions, the net effect of hyperuricemia, particularly if marked and persistent, will affect the renal outcome adversely.

Acute uric acid nephropathy should be suspected in high risk patients who develop oliguric AKI with significantly elevated serum uric acid concentration of more than 10-15 mg/dL and presence of copious uric acid crystals in the urinary sediment [3]. The urinalysis however may be normal if there is no output from the obstructed nephrons [4]. As renal failure is also associated with a rise in serum uric acid as a result of decreased excretion, it may be difficult to determine if hyperuricemia or AKI developed first. Kelton J et al. have proposed that, in adults, a urine uric acid to creatinine ratio of more than 1 is highly suggestive of acute uric acid nephropathy [6]. Some researchers also report that the calculation of uric acid excretion corrected for creatinine greater than 0.57 mg/dL GFR is suggestive of uric acid nephropathy since other forms of AKI would have reduced uric acid excretion [3].

Seizures lead to distinctive metabolic changes depending on the type, length, and intensity of the seizures as well as the patients preexisting condition. Whole body muscle contractions and the activation of the neuroendocrine system to secrete catecholamines increase the cerebral, muscular, and cardiac oxygen demands, while impaired breathing impedes the compensatory mechanisms to satisfy this demand. This causes the irritated muscles to release CK and myoglobin and hypoxic tissues to leak lactate, ammonia, and urea. Afterwards, an inflammatory reaction with cytokine release and leukocytosis occurs [7]. Seizure induced rhabdomyolysis and AKI have been reported [7, 8]. However, seizures have rarely been reported as cause of hyperuricemia and acute uric acid nephropathy.

Acute severe hyperuricemia following recurrent episodes of grand mal seizures or status epilepticus was first reported by Warren et al. in 1975 [9]. None of the patients had significant myoglobinuria nor pigmented granular casts in the urine. All the seven patients reported in the study developed reversible renal failure with two requiring hemodialysis. In 1978, Luhdorf et al. found a significant increase in serum uric acid level in seventeen patients, within 24 hours of two or more grand mal seizures [10]. Of the six patients with severe hyperuricemia only two had developed impaired renal function. A case of seizure induced acute urate nephropathy has been reported in a young male following status epilepticus. This patient developed rhabdomyolysis and anuric renal failure requiring renal replacement therapy despite allopurinol and Rasburicase administration [11]. A case of rhabdomyolysis following status epilepticus with hyperuricemia was also recently reported in young male who developed oliguric renal failure requiring hemodialysis [12].

Increased uric acid production and increased serum uric acid have been noted in people undergoing hard physical exercise in hot climates, largely due to breakdown of endogenous muscle protein. It is possible that similar mechanisms would operate in patients experiencing prolonged or recurrent episodes of seizures [9]. Our patient however developed severe hyperuricemia and acute uric acid nephropathy following recurrent seizures without a dramatic rise in the serum CK level. A possible explanation is that seizures can also cause direct nucleotide breakdown, producing increased levels of adenosine which is then converted by xanthine oxidase in the liver to uric acid [13]. In addition, Grosso et al. found the serum nucleotidase activity to be elevated in patients for several hours after seizures, possibly leading to increased systemic breakdown of adenosine triphosphate and generation of urate [14].

Hypoxia and seizure induced lactic acidosis also impairs the renal tubular secretion of uric acid. Warren et al. postulate that seizure induced hyperuricemia could be due to a combination of overproduction and impaired tubular secretion of uric acid. Saugstad [15] has also proposed that excessive release of hypoxanthine by hypoxic tissues after seizures could be transformed to uric acid by xanthine oxidase thus causing hyperuricemia. As uric acid is also considered an effective antioxidant, it is possible that an elevation in uric acid would be to counteract the oxidative stress during epileptic seizure [16]. Metabolic acidosis which accompanies seizures creates an acidic urine in which uric acid is less soluble causing precipitation of uric acid in the tubular lumen [10]. Dehydration due to profuse sweating and hyperthermia during seizures causes increased tubular water resorption and increased urine uric acid concentration. Renal ischemia due to shunting of blood flow from visceral organs to muscles during seizures may also contribute to the kidney injury [10]. Urine microscopy in our patient also showed occasional granular casts suggesting that uric acid also caused mild tubular injury. Early markers of renal tubular injury like urinary *β*2 microglobulin and N-acetyl-beta-D-glucosaminidase (NAG) were however not measured in our patient.

The pathologic features of acute uric acid nephropathy are reversible [2]. The most efficient therapy for dramatically lowering uric acid is Rasburicase, a recombinant form of urate oxidase, a nonhuman proteolytic enzyme that oxidizes uric acid to allantoin, and a metabolite that is far more soluble than uric acid. Rasburicase is contraindicated in patients with G6PD deficiency (G6PDD) due to risk of drug induced hemolytic anemia [17]. G6PD level was however not checked before administering Rasburicase in our patient. He had a low risk for developing hemolytic anemia due to him being from an ethnicity with low prevalence of G6PDD and absence of any prior history of anemia. He was however closely monitored after Rasburicase administration and tolerated it well. Rasburicase therapy is efficacious in reducing serum uric acid levels with associated diuresis much faster and more effectively than allopurinol, a xanthine oxidase inhibitor [18]. Allopurinol prevents the formation of new uric acid; however, it does not remove the existing uric acid. Moreover, allopurinol also increases the urinary excretion of xanthine which can crystallize and cause kidney injury [2]. Urinary alkalization to pH>6 helps uric acid solubility; however it has not been proven to be an effective therapy in TLS with acute uric acid nephropathy, due to increased calcium phosphate precipitation [2]. Hemodialysis is also quite effective in rapidly reducing uric acid concentrations. Kjellstrand et al. found that oliguria due to acute uric acid nephropathy responds quickly to hemodialysis with initiation of diuresis when serum uric acid concentration falls below 10 mg/dL. Peritoneal dialysis is much less efficient in reducing uric acid concentration [19].

In our patient urine microscopic findings of uric acid crystals lead to early diagnosis of uric acid nephropathy. Timely administration of Rasburicase prevented the need for hemodialysis in this patient and led to complete renal recovery. Our case illustrates the importance of urine microscopy and of monitoring uric acid levels in patients with prolonged seizures and AKI without rhabdomyolysis, as uric acid could be the likely culprit.

## Figures and Tables

**Table 1:** Laboratory data of the patient.

**Time after admission** **(time after Rasburicase administration)**	**Day1**	**Day2**	**Day2** **(0 hrs)**	**Day2** **(4 hrs)**	**Day3** **(12 hrs)**	**Day4** **(24 hrs)**	**Day5** **(48 hrs)**	**Day6**	**Day7**
BUN (mg/dL)	14	29		39	43	55	65	47	29
Creatinine (mg/dL)	1.7	4.9	5.2	6.1	6	5.5	3.1	1.8	1.2
Sodium (mEq/L)	141	140		139	140	139	143	140	
Potassium (mEq/L)	3.7	3.8		3.7	3.5	4.6	4.5	4.4	
CK (U/L)	297	663		464	501	792			
Uric acid (mg/dL)	NA	NA	15	4	0.8	<0.2	<0.2		

*Abbreviations.* BUN: blood urea nitrogen; CK: creatine kinase; NA: not available.
